# Middle-income countries graduating from health aid: Transforming daunting challenges into smooth transitions

**DOI:** 10.1371/journal.pmed.1002837

**Published:** 2019-06-25

**Authors:** Gavin Yamey, Osondu Ogbuoji, Justice Nonvignon

**Affiliations:** 1 The Center for Policy Impact in Global Health, Duke Global Health Institute, Duke University, Durham, North Carolina, United States of America; 2 School of Public Health, University of Ghana, Legon, Ghana

## Abstract

Gavin Yamey and co-authors discuss approaches to providing support for middle-income countries transitioning away from health aid.

In the sustainable development goals (SDGs) era, the global health landscape is undergoing a rapid and profound set of transitions that threaten to stall or even derail progress in health improvement. These shifts are primarily affecting middle-income countries (MICs), where over 70% of the world’s poor now live [1]. Sustaining global health progress will depend on how domestic and international health policymakers and actors navigate 4 transitions facing MICs: shifts in diseases, demography, development assistance for health, and domestic health financing, or the “4Ds” of global health transition.

## The changing nature of disease and demography

The first transition is in disease patterns—the global burden of disease is now shifting to noncommunicable diseases (NCDs) and injuries. In many MICs, this rise in NCDs is happening on the landscape of an unfinished agenda of high mortality from infectious diseases and maternal and child health conditions within subnational pockets of poverty, leading to a double burden of disease. The shift toward NCDs means that these countries must develop health systems that can provide not only episodic care but also long-term, complex healthcare capable of tackling multimorbidity [2]. In addition, the recent global health gains, including those in MICs, could be undercut unless urgent and aggressive action is taken to sharply curtail the global rise in obesity and other NCD risk factors [3–5].

These changing disease patterns are closely linked to a second shift—a multifaceted demographic transition. Ageing populations are placing growing demands on the health and social sectors. At the same time, in many low-income countries (LICs) and MICs, there has been an expansion in the adolescent band of the population pyramid (the “youth bulge”) [6]. This bulge is mainly due to significant success in lowering child mortality rates but not total fertility, which has declined at a much lower rate [7].

In many LICs and MICs, often those with the double burden of infections and NCDs, adolescents now account for around 30% to 40% of the population [8]. They face a range of poorly controlled health threats, including road injuries (the top cause of adolescent death in sub-Saharan Africa), HIV, lower respiratory infections, interpersonal violence, and suicide [8]. In addition, migration related to conflict is affecting the demographic transition, placing a large burden on health services in some MICs such as Jordan (where 1 in 12 people is a Syrian refugee) and Lebanon (where 1 in 6 is a Syrian refugee) [9,10]. Such large influxes of migrants expand the population, exerting pressure on health systems that were often already struggling to provide services to the whole population even before the arrival of refugees [11].

## Moving beyond aid to domestic finance

The third shift is a movement toward a postaid world [12]. Over the next few years, more than a dozen MICs are expected to graduate from multilateral development assistance, i.e., from receiving grants or highly concessional loans from multilateral agencies such as the World Bank, the Global Fund to Fight AIDS, Tuberculosis and Malaria (the Global Fund), and, Gavi, the Vaccine Alliance (Gavi) [13,14]. Such graduation leads to the so-called middle-income dilemma, that “although most of the poor now live in pockets of poverty in middle-income countries and face high mortality rates, these countries are regarded as too rich to qualify for aid” [15]. These MICs have reached, or will soon reach, a national gross domestic product per capita that disqualifies them from receiving aid.

**Fig 1:**
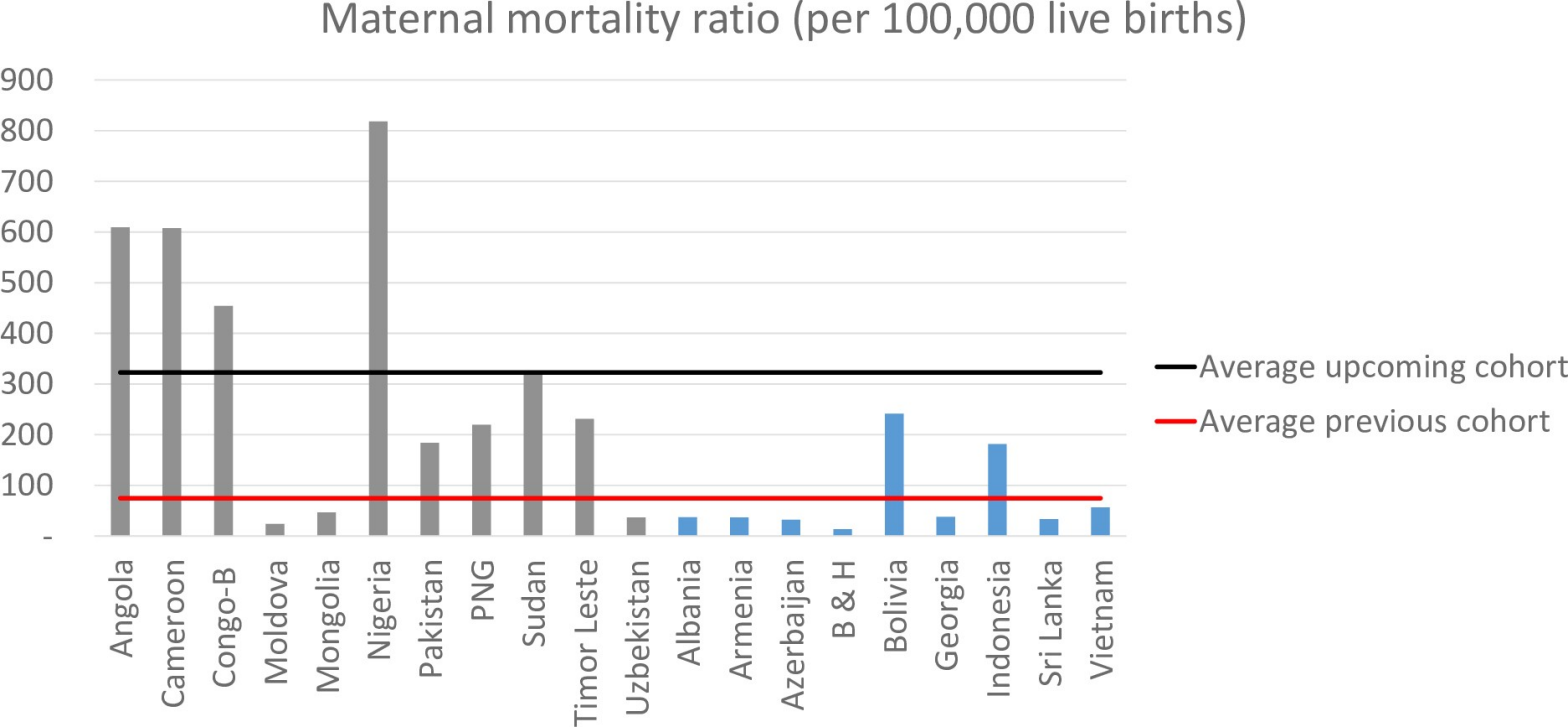
Comparison of the MMR (deaths per 100,000 live births) in the previous cohort of countries versus an upcoming cohort in the period of 5 to 8 years prior to graduation. The previous cohort comprised countries that graduated from IDA between 2010 and 2015 or countries that either graduated or were in the last phases of accelerated transition from Gavi. The upcoming cohort is expected to graduate from IDA, Gavi, or both in coming years (we assumed 2020 as the graduation year). The blue vertical bars represent the previous cohort, and the gray vertical bars represent the upcoming cohort. For each country, an average annual MMR was estimated for this 3-year period prior to graduation. The black horizontal line shows the mean of this value (not weighted for population) across the cohort of upcoming graduates; the red line is the mean across the previous cohort. The average MMR for the upcoming graduates is 323 per 100,000 live births versus 73 per 100,000 live births for the previous cohort. Figure from [13]. B&H, Bosnia and Herzegovina; Congo-B, Congo-Brazzaville; IDA, International Development Association; MMR, maternal mortality ratio; PNG, Papua New Guinea.

How will these upcoming graduate countries cope with this funding cliff? A recent analysis suggests that many will find it tough [13]. The analysis compared 2 groups of countries. The first was a previous cohort of 9 MICs that graduated between 2010 and 2015 from the World Bank’s International Development Association (IDA), which helps the poorest nations, and countries that either graduated or were in the last phases of accelerated transition from Gavi. The second was an upcoming cohort of 11 countries expected to graduate from IDA, Gavi, or both in the coming years. On average, the upcoming cohort has “lower per capita income, greater indebtedness, weaker capacity to efficiently use public resources, more limited and less effective health systems, weaker governance and public institutions, and greater inequality” [13]. To give one stark example, the mean annual maternal mortality ratio of upcoming graduates in the period leading up to graduation is 4 times as high as the average for previous graduates (Fig 1). As seen in the figure, the mean annual maternal mortality ratio is particularly high for 4 upcoming graduates in sub-Saharan Africa: Nigeria, Angola, Cameroon, and Congo-Brazzaville (also known as the Republic of Congo).

Donor exits can create shocks to a country’s health system—affecting human resources for health, service delivery, medicines and technologies, and health financing—especially if both financing and technical support are withdrawn. The upcoming graduates appear to have less domestic capacity to handle such shocks. One particular concern is the impact of such a transition on human resources for health. The loss of external support can lead to gaps in staffing and technical capacity, weakening the health workforce and reducing the quality of health services [16,17]. Another concern is what happens to the quality and coverage of services delivered to vulnerable populations—such as prisoners, men who have sex with men, commercial sex workers, and injection drug users—when donors withdraw support. Such populations are at a higher risk of HIV infection, and prevention and treatment programs for these groups are heavily supported by donors like the Global Fund. When donor exits are poorly managed, these populations can experience HIV resurgence [18].

A fourth, related transition is the new focus within the global health community on domestic resource mobilization for health, exemplified by the United Nations’ Addis Ababa Action Agenda that lays out a roadmap for financing the SDGs [19]. Estimates suggest that it will cost LICs and MICs an additional US$371 billion per year in health spending by 2030 to reach the health-related SDG targets [20]. Most of this additional spending will have to come from domestic sources. Yet many countries have so far seen little or no mobilization of domestic resources, in part because they have not prioritized health spending. Indeed, there is growing evidence that health has become a lesser budgetary priority in many countries [21].

## MICs need a joined-up approach to transition

These complex transitions are highly interlinked and cannot be managed in isolation. To give one example, Ghana, which is currently in Gavi’s preparatory transition phase, cannot only worry about replacing external financing for vaccination; it must also prepare for a rise in the prevalence of NCDs and in health costs, linked to an extraordinary rise in obesity and overweight in its population. More than 4 in 10 Ghanaians now have obesity or are overweight [22]. All MICs need an overarching, joined-up strategic approach to transition. But what would such an approach entail?

First, a transformation of health systems is needed—including benefits packages and delivery platforms—in ways that respond to the shifts in disease burden and demography. Health services will need to be delivered mostly through primary care platforms, which will have to provide “integrated, longitudinal models of care for many chronic health conditions” [23]. The second component of a joined-up approach would be a health financing revolution—involving sustained domestic resource mobilization, pooling, and strategic purchasing of services. Governments of MICs can draw on an expanding evidence base on how to raise revenues for health through tax reform and to allocate and spend funds in ways that maximize health and value for money [24]. Third, joint donor–MIC transition plans must be developed that take into account the importance of health system strengthening and of reaching vulnerable populations before, during, and after transition [18,25]. Finally, the international community can support transitioning MICs through providing global public goods for health. These goods include supporting (i) the development of new health technologies to tackle diseases of poverty, (ii) pooled procurement, market shaping, and revolving funds to bring down the prices of medicines, vaccines, diagnostics, and other health technologies, and (iii) tackling global antimicrobial resistance, including the control of multidrug-resistant tuberculosis [17].

With a joined-up strategy, the 4Ds of global health transition could become an opportunity for accelerated rather than stalled progress. MICs themselves should, of course, be in the driving seat when it comes to leading such strategy development, supported by technical agencies and donors who assist with transition planning and who boost their support for global public goods. By anticipating the disease and demographic trends ahead and what these mean for future financing needs, mobilizing the required resources to fund the right health benefits packages, and supporting intersectoral policies for health improvement (e.g., policies to curb indoor air pollution and road traffic injuries) [26], MICs could see a health transformation in the SDGs era.

## Abbreviations

B&H: Bosnia and Herzegovina
Congo-B: Congo-Brazzaville
IDA: International Development Association
LIC: low-income country
MIC: middle-income country
MMR: maternal mortality ratio
NCD: noncommunicable disease
PNG: Papua New Guinea
SDG: sustainable development goal
4Ds: diseases, demography, development assistance for health, and domestic health financing
